# Contralateral Structure and Molecular Response to Severe Unilateral Brain Injury

**DOI:** 10.3390/brainsci15080837

**Published:** 2025-08-05

**Authors:** Xixian Liao, Xiaojian Xu, Ming Li, Runfa Tian, Yuan Zhuang, Guoyi Gao

**Affiliations:** 1 Department of Neurosurgery, Beijing Tiantan Hospital, Capital Medical University, Beijing 100070, China; xixianliaosmuer149@163.com (X.L.); lmccmu@163.com (M.L.); trftc@126.com (R.T.); 2 Beijing Key Laboratory of Central Nervous System Injury, Beijing Neurosurgical Institute, Capital Medical University, Beijing 100071, China; xjianxu@163.com

**Keywords:** contralateral hemisphere, glia, synapse, transcriptome, unilateral brain injury

## Abstract

**Background**: Severe damage to one side of the brain often leads to adverse consequences and can also cause widespread changes throughout the brain, especially in the contralateral area. Studying molecular changes in the contralateral cerebral hemisphere, especially with regard to genetic regulation, can help discover potential treatment strategies to promote recovery after severe brain trauma on one side. **Methods**: In our study, the right motor cortex was surgically removed to simulate severe unilateral brain injury, and changes in glial cells and synaptic structure in the contralateral cortex were subsequently assessed through immunohistological, morphological, and Western blot analyses. We conducted transcriptomic studies to explore changes in gene expression levels associated with the inflammatory response. **Results**: Seven days after corticotomy, levels of reactive astrocytes and hypertrophic microglia increased significantly in the experimental group, while synapsin-1 and PSD-95 levels in the contralateral motor cortex increased. These molecular changes are associated with structural changes, including destruction of dendritic structures and the encapsulation of astrocytes by synapses. Genome-wide transcriptome analysis showed a significant increase in gene pathways involved in inflammatory responses, synaptic activity, and nerve fiber regeneration in the contralateral cortex after corticorectomy. Key transcription factors such as NF-κB1, Rela, STAT3 and Jun were identified as potential regulators of these contralateral changes. Quantitative reverse transcription polymerase chain reaction (qRT-PCR) confirmed that the mRNA expression levels of Cacna1c, Tgfb1 and Slc2a1 genes related to STAT3, JUN, and NF-κB regulation significantly increased in the contralateral cortex of the experimental group. **Conclusions**: After unilateral brain damage occurs, changes in the contralateral cerebral hemisphere are closely related to processes involving inflammation and synaptic function.

## 1. Introduction

Although severe brain injury accounts for approximately 10% of brain injuries [1], it is associated with high mortality; however, available effective treatments are limited [2]. Unilateral brain injury can manifest in varying degrees of severity, contingent upon the nature and intensity of the trauma sustained. The resultant brain lesions may span a spectrum, ranging from minor infarctions to extensive cortical loss. Individuals who survive severe brain injuries often face limited prospects for functional recovery and may endure permanent disabilities, particularly in cognitive, motor, and sensory domains. Given the extensive connectivity between the brain hemispheres, focal brain injury can elicit a diffuse response that extends well beyond the immediate injury site. However, the heterogeneous nature of the regional response to injury is seldom thoroughly investigated. While numerous studies have documented changes in the ipsilateral hemisphere following severe unilateral brain injury, alterations in the contralateral hemisphere remain underexplored and may hold significant potential as a therapeutic target in clinical settings.

Severe brain injury is a highly complex pathological process. The initial mechanical impact is followed by a cascade of molecular reactions, which subsequently trigger inflammation and the release of various biochemical mediators. These mediators can profoundly influence neural circuits, leading to secondary injury and long-term functional deficits [3,4,5]. Following unilateral brain injury, inflammation initially manifests in the injured hemisphere, engaging a complex molecular cascade response that involves multiple cell types, including microglia and astrocytes [6,7]. These reactive cells, including microglia and astrocytes, proliferate and undergo significant morphological changes in response to injury. They subsequently secrete a variety of cytokines and chemokines, which play crucial roles in modulating the inflammatory response and influencing the overall pathological process [8,9]. Ultimately, these cellular and molecular changes can profoundly influence the brain microenvironment and synaptic transmission, thereby affecting neural function and contributing to long-term outcomes following brain injury. Zhang et al. [10] showed that microglial complement receptor 3 triggered long-term depression of synaptic transmission in the ipsilateral hippocampus, which contributed to post-traumatic cognitive impairment. Krukowski et al. [11] demonstrated microglia activation and accumulation of C1q that paralleled synapse loss in the ipsilateral hippocampus 30 days after traumatic brain injury. Additionally, Perez et al. [12] reported that the selective elimination of astrocytic D-serine by astrocytes themselves could enhance synaptic plasticity following traumatic brain injury (TBI). Moreover, the upregulation of astrocyte-related ephrin B1 is concomitant with a decrease in vGlut1-positive glutaminergic synaptic input within the ipsilateral hippocampus [13]. However, the elevation of THBS1 and Hevin, which are secreted by astrocytes, plays a crucial role in promoting post-injury synaptic reorganization within the ipsilateral hemisphere [14,15].

Both hemispheres maintain a degree of independence while being unified in integrating information and executing functions through synaptic remodeling. In our previous study, we reported that contralateral synapses tend to mature over time following injury, and we observed abundant synaptic proliferation at day 7. The injury-induced contralateral response, which involves inflammatory processes, may significantly influence this synaptic remodeling and maturation. However, the extent of this influence has been historically underestimated [16,17,18]. Elucidating the molecular and genetic changes in the contralateral hemisphere may provide a foundation for developing therapies aimed at enhancing recovery from severe brain injury. The study involved resection of the right motor cortex in a rat model to simulate extensive unilateral brain injury. The purpose is to study the molecular and transcriptome changes that occur in the contralateral motor cortex during the subacute phase of recovery.

## 2. Materials and Methods

### 2.1. Animals

In this study, twenty-eight adult male Sprague Dawley rats weighing between 180 g and 200 g were obtained from Beijing Vitonglihua Experimental Animal Technology Co., Ltd. (Beijing, China). The study was approved by the Beijing Institute of Neurosurgery Animal Welfare and Ethics Committee with the approval number 202003005. The entire research process strictly follows the 3Rs principle, namely, substitution, reduction, and optimization. The animals were housed in a standardized environment with four to five rats per cage under a twelve-hour light/twelve-hour dark cycle. Environmental parameters remained in the temperature range of 22 degrees Celsius plus or minus two degrees Celsius, with a humidity of 50% to 60%. They had unlimited access to food and water at all times.

### 2.2. Right Motor Cortex Resection

The rats were randomly divided into two groups: a control group (sham-operated group) consisting of 14 animals and an experimental group, also consisting of 14 rats. The rats used in the experiment had their right motor cortex surgically removed and monitored for seven days. Anesthesia was initially performed with 5% isoflurane (provided by Shenzhen Rayward Life Technology Co., Ltd., Shenzhen, China), which was mixed with pure oxygen at a flow rate of 1 L per minute and continued until the animal lost the ability to turn on its own. Anesthesia was subsequently maintained with 2% isoflurane (Sigma product, St. Louis, MO, USA). The rats were then placed in a prone position, and a median incision was made along the scalp to expose the skull. The coordinates for the craniotomy were reported in our previous study as follows [19]: The anterior position is 4 mm anterior to the anterior fontanel point, while the posterior point is 3.5 mm posterior to the anterior fontanel point. The medial reference point is 0.5 mm lateral to the sagittal suture, and a thin bone layer is retained above the sagittal sinus. The area corresponding to the lateral landmark is close to the attachment of the temporal muscle. We performed the craniotomy using a Cranial Drill from RWD Life Science Co., Ltd. (Shenzhen, China), carefully retaining the underlying dura mater. With the help of a surgical microscope, a C-shaped incision was made in the dura so that the motor cortex could be resected to a depth of 1.5 mm with a microsurgical needle, using the corpus callosum as the lower limit of resection. After hemostasis was completed, the dura mater was repositioned and not sutured, and the skin was sealed with 4-0 nylon sutures. After the animals regained consciousness from anesthesia, they were returned to their original cages.

### 2.3. Magnetic Resonance Imaging (MRI)

Twenty-four hours after resection of the right motor cortex, magnetic resonance imaging scans were performed using a 7.0 Tesla system (product of Bruker AG, Mannheim, Germany) equipped with four-channel coils specifically for brain imaging in rats. The animals were anesthetized with 10% chloral hydrate during imaging. T2-weighted images were acquired using the following parameter settings: a repetition time of 3380 milliseconds, an echo time of 41 milliseconds, a matrix size of 192 × 320 pixel, a total of 18 slices, a field of view of 24 × 30 mm, and a slice thickness of 0.5 mm, averaging twice per scan.

### 2.4. RNA Extraction

Seven days after the cortex was removed, the rats were deeply anesthetized and perfused with ice-cold 0.9% physiological saline and then their brains were carefully removed, and left motor cortex tissue located approximately 2 mm from the midline was collected onto a cooled stainless steel plate, using Trizol reagent (Thermo Fisher Technologies, Waltham, MA, USA, product number 15596018). Total RNA was isolated according to the protocol provided by the manufacturer, and the isolated RNA was then measured, purified, and evaluated using a Bioanalyzer 2100 system equipped with the RNA 6000 Nano LabChip Kit (Agilent Technologies, Santa Clara, CA, USA, product number 5067-1511).

### 2.5. mRNA Library Construction and Sequencing

Only RNA samples with an RNA Integrity Value (RIN) greater than 7.0 were selected for the preparation of sequencing libraries. The process starts with extracting mRNA from 5 micrograms of total RNA using Dynabeads Oligo (dT) (Thermo Fisher Technologies) and performing two rounds of purification. The purified mRNA was then cut into smaller fragments and reverse transcribed into complementary DNA (cDNA) using SuperScript II reverse transcriptase (InviTech, Waltham, MA, USA, product number 1896649) to build a cDNA library. The average insert size of the resulting library was approximately 300 base pairs, and the error was within the range of plus or minus 50 base pairs. Sequencing was carried out on the Illumina Novaseq 6000 system with a double-end read of 150 base pairs each (Hangzhou Liebing Biomedical Technology Co., Ltd., Hangzhou, China), completed according to the manufacturer’s recommendation.

### 2.6. Data Processing and Analysis

High-quality reads were generated by filtering out raw reads containing adapters, primers, and nucleotides with quality scores below 20. The quality of the filtered reads was assessed using FastQC (http://www.bioinformatics.babraham.ac.uk/projects/fastqc, accessed on 8 Februry 2022). Subsequently, the clean reads were aligned to the rat reference genome using the HISAT2 package (v2.1.0, https://ccb.jhu.edu/software/hisat2/index.shtml, accessed on 8 July 2025). The aligned reads were then assembled using StringTie (gffcompare-0.9.8, http://ccb.jhu.edu/software/stringtie, accessed on 15 Februry 2022) with default parameters, and the resulting assemblies from all samples were merged to construct the final transcriptomes. The expression levels of transcripts and the abundance of mRNA (measured in fragments per kilobase of transcript per million mapped reads) were quantified using StringTie and ballgown (http://ccb.jhu.edu/software/stringtie/gffcompare.shtml, accessed on 23 Februry 2022, version: gffcompare-0.9.8).

### 2.7. Differentially Expressed Gene (DEG) Analysis

Differential expression analysis of gene expression differences between the control group (sham operation group) and the experimental group was performed using DESeq2 software (v1.34.0). In addition, the data was also analyzed using edgeR software (v3.36.0) for comparison. When the false discovery rate (FDR) of a gene was less than 0.05 and the absolute fold change was at least 2, it was classified as a differentially expressed gene.

### 2.8. Gene Ontology (GO) and Kyoto Encyclopedia of Genes and Genomes (KEGG) Pathway Enrichment Analysis

The role of differentially expressed genes was clarified through Gene Ontology (GO) analysis (http://geneontology.org, accessed on 8 April 2022 ) and Kyoto Encyclopedia of Genes and Genome (KEGG) pathway analysis (http://www.genome.jp/kegg, accessed on 17 April 2022). The ClusterProfiler R software package (version 4.12.0) was used for enrichment analysis of GO terms and KEGG pathways, with annotations derived from a rat-specific database. Genes were selected based on the false discovery rate (FDR) method, and both the *p*-value and the corrected *p*-value threshold were set below 1. In GO analysis, the focus is on the biological process (BP) and molecular function (MF) categories. *p* < 0.05 indicated significant enrichment.

### 2.9. Gene Set Enrichment Analysis (GSEA)

Gene Set Enrichment Analysis (GSEA) was performed to identify genes with significant differences within specific KEGG pathways. Using the “Combine Data Sets into Gene Symbols” option in GSEA and an appropriate remapping chip (rat_Gene_Symbol_Remapping_MSigDB.v7.0.chip), the rat gene was converted to the corresponding human orthologue gene. The GSEA tool was then used to assess the enrichment of classical pathways, focusing on the set of C2 KEGG pathways. When the normalized enrichment fraction (|NES|) is above 1, the nominal *p*-value is below 0.05, and the false discovery rate (FDR) q-value is below 0.25, significant enrichment of the channel is considered.

### 2.10. Transcription Factor (TF) Analysis and Hub Genes with Protein–Protein Network Analysis

Trrust (v2.303.2, https://www.grnpedia.org/trrust/Network_search_form.php, accessed on 6 May 2022) was used to explore the association between transcription factors (TFs) and pathway-related genes that showed upregulation as determined by GSEA (significance thresholds were set at *p* < 0.05 and FDR q-values < 0.1). For each gene-transcription factor combination, Trrust evaluates whether the transcription factor is known or predicted to bind to the gene, which is supported by evidence in the scientific literature (consider *p* < 0.05 and FDR q-values < 0.25). We built a protein–protein interaction (PPI) network using data from the online Neighboring Gene Duplicate Instance Search Tool (STRING, v2.54) database (http://string-db.org, accessed on 18 May 2022), which compiles identified and computationally predicted protein interactions. The interaction data was then imported into Cytoscape (v3.7.2) for visualization and further analysis of the molecular interaction network. In order to accurately locate key genes, the Cytohubba plug-in Cytoscape was used to identify the hub gene with the most connections through the node degree method.

### 2.11. Golgi Staining

The rats were decapitated, and their brains were removed 7 days after injury. After washing with Milli-Q water for 30 min, Golgi staining of brain tissue was performed using the FD Rapid Golgi Stain™ Kit (FD Neuro Technologies, Inc., Columbia, MD, USA) according to the manufacturer’s instructions [20]. Tissue sections were examined using an optical microscope (Axio Imager M2 from Zeiss, Oberkohen, Germany), and dendrites were observed through a 10× objective.

### 2.12. Electron Microscopy (EM)

After the rats were anesthetized, they were perfused with physiological saline, and samples were collected from the left motor cortex. The collected brain tissue was stored in 2% glutaraldehyde solution containing 0.1M phosphate buffer and then treated with 1% osmium tetroxide in the same buffer. Tissue specimens were gradually dehydrated with varying increasing concentrations of ethanol, followed by infiltration with propylene oxide, and then embedded in Spurr epoxy resin. For microscopic observation, specimens were ultra-thin sectioned using an ultra-thin microtome (Leica CM1950, Wetzlar, Germany), and then the sections were stained with uranium acetate and lead citrate dyes. The quantification of synapses around astrocytes was performed using ImageJ software (version 1.53a). Statistical comparisons between groups were conducted using a *t*-test, and the significance threshold was set at *p* < 0.05.

### 2.13. Immunofluorescence Staining

After perfusion with normal saline and paraformaldehyde, brain tissue should be stored in 4% paraformaldehyde for at least 24 h. The samples were then dehydrated in 30% sucrose for three days and then cut into 10 micron crown sections using a cryogenic microtome (CM1950, Leica AG Wetzlar, Wetzlar, Germany). Prior to staining, brain sections were subjected to heat-induced antigen retrieval via microwave treatment in 10 nM sodium citrate buffer at pH 6.0. It was then blocked with a solution containing 10% normal goat serum and 0.3% Triton X-100 for one hour. Sections were incubated overnight with primary antibodies targeting (GFAP; 1:500, Mouse, cat # ab279290, Abcam, Cambridge, UK); Iba1 (1:500, Rabbit, cat # 019-19741, Wako, Japan); Synapsin-1 (1:1000, Rabbit, cat # ab254349, Abcam, Cambridge, UK); and neuronal nuclei (NeuN, 1:500, Mouse, cat # ab104224, Abcam, Cambridge, UK). The next day, a secondary antibody conjugated to Alexafluor 488 or 594 (1:500, Abcam, Cambridge, UK) was applied for one hour to detect primary antibody binding. In order to visualize the cell nucleus, samples were sealed with a Vectashield sealing tablet containing DAPI (ab104139, Abcam, Cambridge, UK). Fluorescence images were acquired with an Axio Imager M2 microscope (Zeiss, Oberkohen, Germany) with standardized exposure times, with Iba1 of 360 milliseconds, GFAP of 320 milliseconds, Synapsin-1 of 350 milliseconds, NeuN of 140 milliseconds, and DAPI of 4.4 milliseconds, and analyzed using ImageJ software. Statistical comparisons between groups were conducted using a *t*-test, and the significance threshold was set at *p* < 0.05.

### 2.14. Quantitative Reverse Transcription Polymerase Chain Reaction (RT-PCR)

Total RNA was extracted from the left motor cortex (side opposite the resection site) of both groups of rats using Trizol reagent (Biosharp, cat. BS259A, Tallinn, Estonia) according to the manufacturer’s protocol. The RNA isolated from each sample was subsequently reverse transcribed into complementary DNA (cDNA) using SuperScript II reverse transcriptase. For quantitative PCR (qPCR), the reaction system was set to 20 µL containing SYBR Premix Ex Taq (Thermo Fisher, cat 4367659). Each sample was analyzed in triplicate to ensure accuracy. Cycle threshold (Ct) values were recorded, and the relative content of target gene mRNA was determined using the 2^−ΔΔCT^ method. These expression levels were normalized to β-actin expression in all samples. The fold change in the transcription level of the target gene was calculated relative to the sham-operated control group. To determine statistically significant differences in gene expression, a *t*-test was performed, and the significance threshold was set at *p* < 0.05. The primer sequences used to amplify the target gene are listed below:
Cacna1c:forward 5′-AAGCGGCAGCAGTATGGGAAAC-3′,
reverse 5′-TCAGAGTCAGGCAGAGCAGAGC-3′Slc2a1:forward 5′- CATCCACCACACTCACCACACTC-3′,
reverse 5′-GCCTGCCAAAGCGATTAACAAAGAG-3′Tgfb1:forward 5′- GACCGCAACAACGCAATCTATGAC-3′,
reverse 5′-CTGGCACTGCTTCCCGAATGTC-3′β-Actin:forward 5′- TGTCACCAACTGGGACGATA-3′,
reverse 5′- GGGGTGTTGAAGGTCTCAAA-3′

### 2.15. Western Blot (WB) Analysis

The contralateral motor cortex tissue was collected for Western blot analysis using gradient centrifugation, as previously described [21]. Protein levels were quantified using the bisquinolinic acid (BCA) assay, and 30 micrograms of extracted protein was loaded on sodium dodecyl sulfate–polyacrylamide gel electrophoresis (SDS-PAGE) for separation. After electrophoresis, the protein was transferred to a polyvinylidene fluoride (PVDF) membrane. These membranes were blocked with 5% skimmed milk for one hour at room temperature and then incubated overnight at 4 °C with primary antibodies, which included Synapsin-1 (1:1000, Rabbit, cat#ab254349, Abcam, Cambridge, UK); PSD-95 (1:1000, Mouse, cat No.MA1-045, Invitrogen, Carlsbad, CA, USA); IL-1β (1:1000, Rabbit, cat No. 26048-1-AP, proteintech, Wuhan, China); TGF-β (1:1000, Rabbit, cat#3711S, Cell Signaling TECHNOLOGY, Danvers, MA, USA); and IL-6 (1:1000, Rabbit, cat GB11117, Servicebio, Wuhan, China). Subsequently, the membranes were incubated with the respective secondary antibodies (diluted 1:5000) for one hour at room temperature. Visualization of protein bands was achieved through chemiluminescent detection using the Amma Imager 600 system (GE Healthcare, Boston, MA, USA). Optical density analysis was performed using ImageJ software (version 1.53a) and band intensity normalized to β-actin levels. Statistical comparisons were performed using a *t*-test, and *p*-values less than 0.05 were considered statistically significant.

## 3. Results

### 3.1. MRI Evaluation of the Model

**Figure 1:**
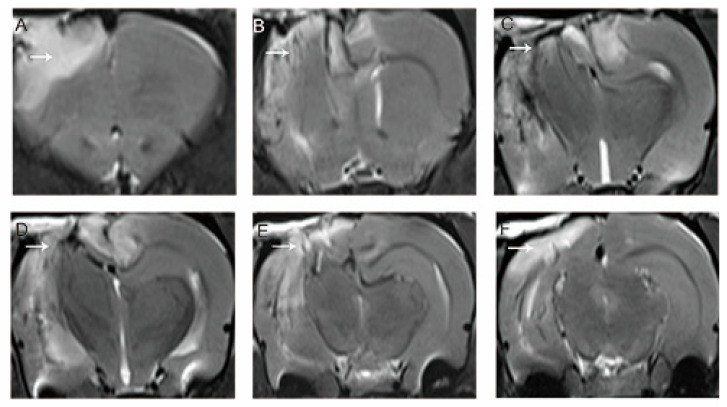
T2-weighted imaging of the brain 1 day after right motor cortex resection. (**A**–**F**) Lesions on different sections from anterior to posterior (white arrows). Edema appeared at the resection site and extended to subcortical areas, including the hippocampus and thalamus. In contrast, the structure of the contralateral hemisphere was intact; however, a high T2 signal representing hemorrhage was obvious around the midline.

Figure 1 shows an MRI scan taken the day after the right motor cortex resection. The lesion extends along the anteroposterior axis from the olfactory bulb to the hippocampus. The T2-weighted image shows irregular signal intensity in the damaged area, reflecting the presence of swelling and bleeding. Extensive edema affects subcortical structures such as the corpus callosum, hippocampus, and thalamus. In contrast, the contralateral (left) cerebral hemisphere appears structurally normal. However, signs of bleeding characterized by an elevated T2 signal were observed around the midline.

### 3.2. Right Motor Cortex Resection Resulted in Structural and Molecular Changes in the Contralateral Motor Cortex

Although no significant visible magnetic resonance imaging changes in the contralateral motor cortex were observed after corticorectomy, slight molecular and structural changes were observed in this area seven days after injury.

**Figure 2:**
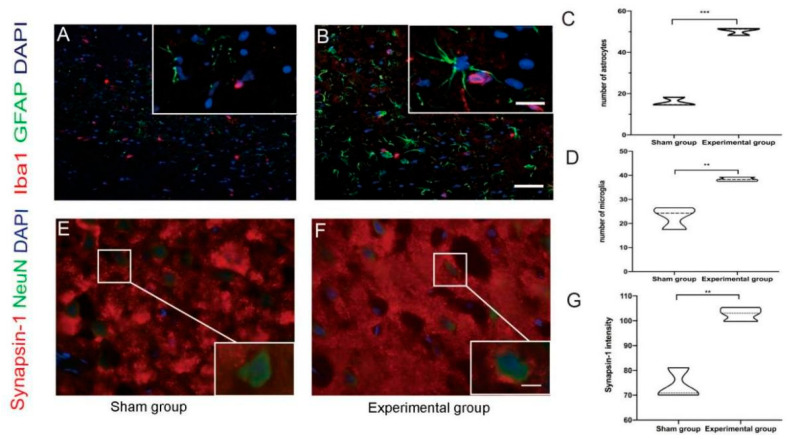
GFAP, Iba1, and synapsin-1 immunofluorescence in the contralateral motor cortex. (**A**,**B**) Astrocytes and microglia in the sham and experimental groups, respectively; scale bar, 50 µm. On the top right, the morphology of astrocytes and microglia is specifically represented; scale bar, 20 µm. (**C**) The number of active astrocytes in the contralateral motor cortex was higher in the experimental group than in the sham group (*n* = 6; *** *p* = 0.001). (**D**) Compared with the sham group, the number of microglia in the experimental group was also elevated (*n* = 6; ** *p* = 0.005). (**E**,**F**) Expression of synapsin-1 in layer V of the motor cortex in the sham and experimental group; scale bar, 20 µm; the square shows synapsin-1 expression around one neuron; scale bar, 5 µm. (**G**) The expression of synapsin-1 in layer V of the left motor cortex was slightly higher in the experimental group (*n* = 6; ** *p* = 0.002).

The main glial cells in the central nervous system, resident astrocytes and microglia, are the main responders to nerve injury. In the ipsilateral and contralateral motor cortex areas, the number of reactive glial cells in the experimental group increased significantly compared to the sham-operated group. Specifically, in the contralateral motor cortex, more activated astrocytes appeared in the experimental group. These cells were enlarged and hypertrophic in shape, with stretched processes and swollen cell bodies, and at the same time, glial fibrillary acidic protein expression levels also increased (Figure 2A,C). These groups showed similar differences in microglia activity and Iba1 expression in the contralateral motor cortex (Figure 2B,D). One week after the corticotomy was performed, the levels of IL-1β and TGF-β in the fifth layer of the contralateral motor cortex in the experimental group increased compared with the sham operation group. On the contrary, IL-6 expression did not show significant differences between the two groups (Supplemental Figure S1A,C).

Synapsin-1 is a phosphoprotein found in synapses. It is associated with synaptic vesicles and is located mainly at the presynaptic terminal, and it plays a role in the release of neurotransmitters. Seven days after resection of the cerebral cortex, levels of synapsin-1 in the fifth layer of the contralateral motor cortex were higher in the experimental group than in the control group that received sham surgery (Figure 2E–G; Supplemental Figure S1A,B).

PSD-95 is a scaffold protein that is highly enriched in the postsynaptic membrane. It belongs to the membrane-associated guanylate kinase family. It maintains synaptic structure and function and participates in synaptic signaling and plasticity regulation by mediating protein interactions. Similar to Synapsin-1, seven days after resection of the cerebral cortex, levels of PSD-95 in the fifth layer of the contralateral motor cortex were also higher in the experimental group than in the control group that received sham surgery (Supplemental Figure S1A,D).

**Figure 3:**
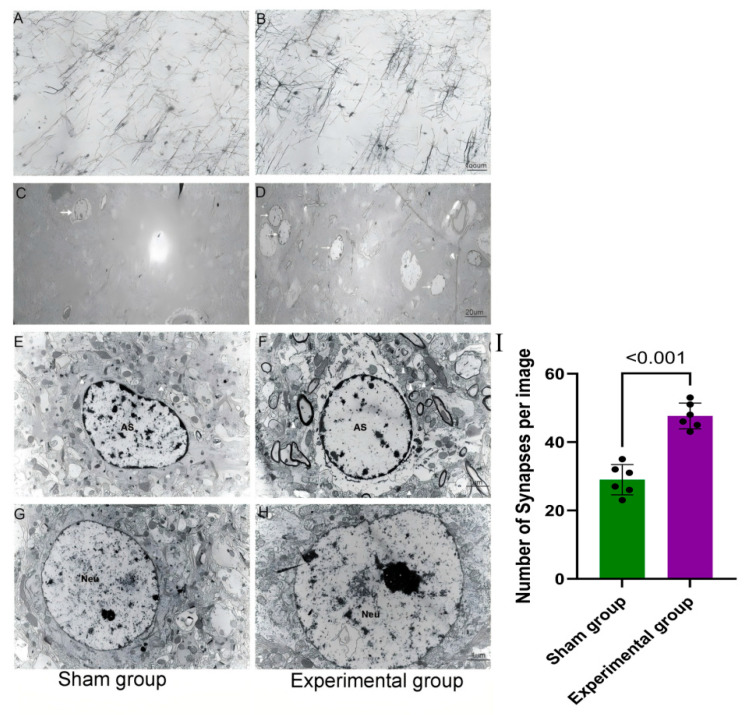
Golgi staining and EM examination of the contralateral motor cortex. Golgi staining of the contralateral motor cortex in the sham (**A**) and experimental groups (**B**). Dendrites in Layers III–IV were observed; scale bar, 100 µm. In the sham group, layered neurons were detected, and the dendrites were regularly arranged. In the experimental group, neurons with short dendrites and messy dendrite arrangements were observed. (**C**–**H**) Electron microscope images of the contralateral motor cortex in the sham and experimental groups (AS, astrocyte; EM, electron microscope; Neu, neuron). (**C**) The small number of astrocytes in the sham group (white arrow); scale bar, 1 µm. (**D**) The number of astrocytes increased in the contralateral motor cortex 7 days after right cortical resection (white arrows); scale bar, 1 µm. (**E**) Astrocytes in the sham group had normal morphology and were accompanied by many synapses (white triangles); scale bar, 1 µm. (**F**) Astrocytes in the experimental group showed edema in the cytoplasm and hypertrophic nuclei. More synapses were observed around astrocytes, which represented the shortened distance between the astrocytes and synapses; scale bar, 1 µm. (**G**) Neurons in the sham group had normal morphology with round nuclei and clear chromatin; scale bar, 1 µm. (**H**) Neurons in the experimental group had normal morphology, but their nuclei were slightly hypertrophic; scale bar, 1 µm. (**I**) The number of synapses around astrocytes in the sham surgery group and the experimental group on day 7 (*n* = 6, *p* < 0.001).

Seven days after corticorectomy, changes at the molecular level led to observable microscopic changes in the contralateral motor cortex of the subject. Notably, the dendritic structure in this area appeared disorganized, and the incidence of shorter dendrites increased significantly (Figure 3A,B). Electron microscope observation showed an increase in the number of astrocytes in the contralateral motor cortex, accompanied by swelling of the cytoplasm and nuclei (Figure 3C–F). Synapses were also observed near astrocytes, emphasizing the close connection between astrocytes involved in the injury response and synaptic remodeling (Figure 3E,F,I). Minor changes in the neuron structure were detected, such as slight enlargement of the cell nucleus and an increase in the overall size of the cell (Figure 3G,H).

### 3.3. Gene Expression Changed in the Contralateral Motor Cortex 7 Days After Right Motor Cortex Resection

**Figure 4:**
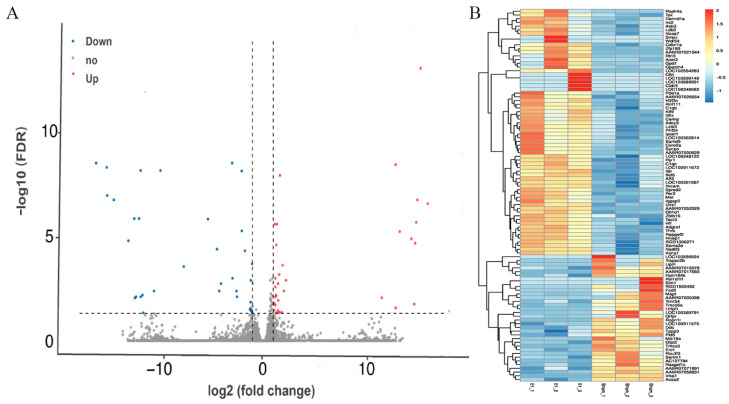
Analysis of differentially expressed genes (DEGs) between the sham and experimental groups. (**A**) A volcano plot of DEGs in the experimental group compared with the sham group. The downregulated, unchanged, and upregulated genes in the contralateral motor cortex after cortical resection are identified by the blue, gray, and red dots, respectively. (**B**) A heatmap of DEGs between the sham group and the experimental group. The color scales stand for significant levels of DEGs. (E: Experimental group).

To investigate how unilateral brain injury affects the contralateral unaffected motor cortex, we compared gene expression profiles in the sham operation group and the experimental group. The analysis showed significant expression differences in 70 of the total 21,106 genes tested. Among the total, 35 genes showed increased expression, while the other 35 genes showed decreased expression levels. Genes with elevated expression are associated with cell adhesion and tight junction formation (e.g., Cldn5 and Cttn), axon guidance (including Met and Sema3e), and also with synaptic activity-related functions (Adcy9) and signaling pathways (Tacr3 and Deptor). In contrast, genes with reduced expression were associated with oxidative stress (Mapk7, Mapt, and Ngfr) and apoptosis (Erc1). The DEG analysis is shown in Figure 4.

### 3.4. Enriched Pathways in Gene Set Enrichment Analysis (GSEA)

**Figure 5:**
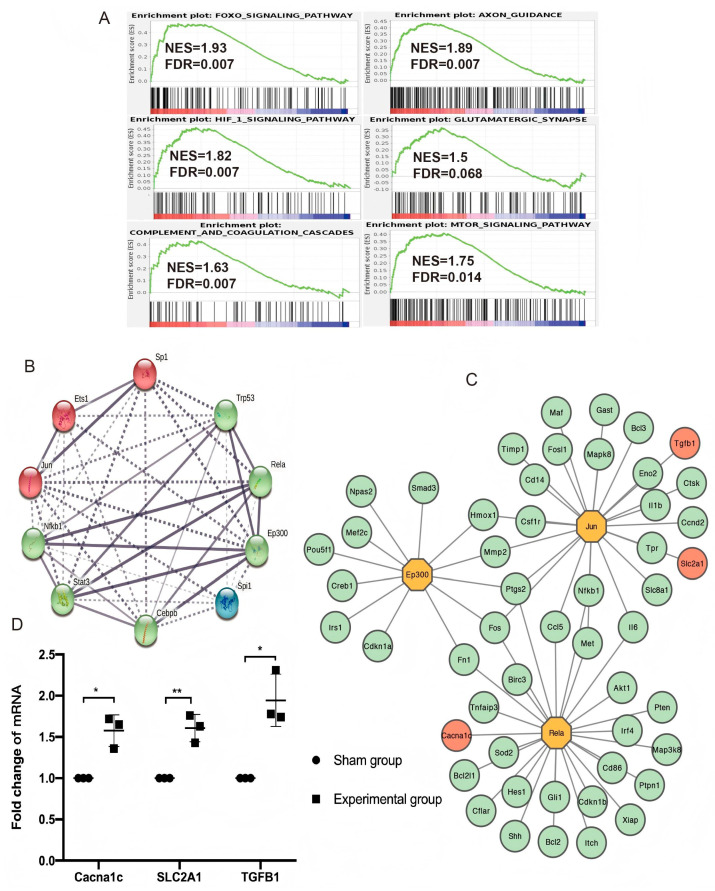
Gene set enrichment analysis (GSEA)and transcription factor (TF) regulatory network analysis (**A**) GSEA enrichment plots of significantly upregulated gene sets in the experimental group compared with the sham group. The enrichment score represented the degree to which the genes in the gene set were overrepresented at the extremes of a ranked gene list. (**B**) The 10 top overlapped TFs are shown with protein–protein interaction (PPI). (**C**) PPI with hub TFs and the genes they regulate (TFs are shown with yellow nodes). Tgfb1 and Slc2a1, which are related to astrocyte function, were regulated by Jun. Cacna1c, which is related to synaptic function, was regulated by Rela (genes are labeled with red circle nodes). (**D**) Quantitative reverse transcription polymerase chain reaction was conducted to verify gene transcript levels. Cacna1c, Slc2a1, and Tgfb1 were upregulated 7 days after right motor cortex resection (*n* = 6; * *p* = 0.035 for Cacna1c; ** *p* = 0.003 for Slc2a1; * *p* = 0.036 for Tgfb1).

Because GO and KEGG pathway analyses rely on identifying differentially expressed genes (DEGs) and are based on pre-set critical criteria, subtle differences between the control and experimental groups may be missed. Therefore, a gene set enrichment analysis (GSEA) was performed, and the results showed significant enrichment in 108 pathways. Pathways that show enhanced activity are mainly involved in inflammatory processes, including the FoxO pathway, the Hif1 pathway, and the complement cascade. There was a significant increase in the expression of genes related to processes such as synaptic activity and axon repair, including glutamatergic synapses, axon guidance, and the mTOR signaling pathway (Figure 5A).

### 3.5. TF Regulatory Network Analysis and Hub Gene Identification

TFs are proteins embedded into protein–protein interaction (PPI) networks that modulate a specific transcriptional response to regulate gene expression at different spatial and temporal levels. They also play a vital role by acting as communication hubs and recruiting protein partners [22]. We predicted key TFs using genes enriched in the upregulated pathways with an FDR value < 0.1 and *p* < 0.05. 158 significant TFs were identified, and 50 overlapped with more than five genes. The top 10 significant TFs with the most overlapped genes were Nfkb1, Sp1, Trp53, Rela, Jun, STAT3, Ets1, Cebpb, Spi1, and Ep300. In addition, the most crucial hub TFs according to the PPI network were Ep300, Rela, and Jun (Figure 5B).

We then inferred a PPI network based on the hub TFs and the genes they regulated. Tgfb1 and Slc2a1 (regulated by Jun) and Cacna1c (regulated by Rela) were identified, which are related to astrocytes and synapses. Detection via reverse transcription polymerase chain reaction (RT-PCR) showed that the expression of messenger ribonucleic acid (mRNA) of Cacna1c, Tgfb1, and Slc2a1 in the contralateral cortex in the experimental group increased (Figure 5C,D).

## 4. Discussion

In our earlier studies, we mainly recorded changes in contralateral synapses after severe unilateral brain injury [19]. Despite this, our understanding of the cellular and molecular processes involved in initial and subsequent severe brain injuries is limited. A deeper understanding of these mechanisms is crucial to developing effective treatment strategies. Secondary injuries, unlike primary injuries at the time of the initial trauma, can occur at any time from minutes to months after the trauma occurs and can last for many years. Our study shows that unilateral damage triggers extensive genetic modification throughout the system, although the corresponding changes at the protein level are relatively small. Transcriptome analysis has emerged as a sensitive method to detect these changes [23,24,25,26]. Seven days after the injury, unilateral brain damage caused by the removal of the right motor cortex triggered the activation and proliferation of microglia and astrocytes, while at the same time increasing the expression of synaptic proteins in the contralateral hemisphere, and genes related to inflammatory responses and synaptic remodeling were also upregulated.

Severe brain trauma can quickly trigger biological changes involving multiple mechanisms, such as inflammatory responses, metabolic regulation, and tissue repair. With the development of transcriptome technology, specific genes responsible for these processes have been accurately identified and targeted. Treatment provides a promising avenue. After a stroke, RNA sequencing showed an increase in genes associated with the TREM-1 and NLRP3 pathways, including Nfkb2 and IL-1β, which contribute to the neuroinflammatory response immediately after ischemia [27]. Interestingly, in a mouse stroke model, clearance of dead cells by macrophages was regulated by PPARγ and STAT6, which also triggered the regeneration of the area around the infarction [28]. Following traumatic brain injury, levels of neuronal and synaptic indicators decline, while markers related to astrocytic hyperplasia, immune cell activation, and cellular stress show increased expression. In mouse models of traumatic brain injury, both microglia and astrocytes showed sustained upregulation of genes associated with the type I interferon pathway and MHC class I molecule presentation [29]. During the chronic phase, expression of genes associated with the complement system, specifically C2, C3, and C4 genes, continues to increase on the same side of the brain [30]. The authors of this study observed elevated levels of cytokines in the contralateral motor cortex of experimental subjects, specifically interleukin-1β (IL-1β) and transforming growth factor-β (TGF-β), as well as increased levels of Synapsin-1 and PSD-95, two kinds of protein associated with synapses. The effect of inflammation on synapse structure seems to be twofold.

Studies have shown that in mouse stroke models with different pathological characteristics, the proliferation of both reactive microglia and astrocytes helps regulate synaptic phagocytosis [31]. Inhibiting this process is expected to prevent synaptic degeneration, which will lead to enhanced neurobehavioral function and reduced brain damage. However, astrocytes are crucial for the formation, growth, and fine regulation of neural networks [32,33]. By releasing proteins and lipid compounds, they promote the development of new synaptic connections and assist in the maturation process of existing synapses. Astrocytes influence the development and maintenance of synapses within their range of action by employing contact-dependent signaling pathways, and they also help clear unnecessary synapses through phagocytosis. A lack of microglia membrane protein TMEM59 impedes microglia’s ability to engage in synaptic phagocytosis, which undermines their ability to devour excitatory synapses. This interference can lead to an imbalance between excitement and inhibition in neuronal activity, contributing to autism spectrum disorders in affected individuals [34]. Therefore, astrocytes and microglia both promote neuron survival and cause neuron damage. Next, our research will explore these functions more deeply by using targeted cell-specific suppression techniques or gene knockout methods.

Many early studies used the contralateral hemisphere as a control point when exploring the effects of brain injury on the affected side, but as genomic techniques continued to develop, the effects of trauma on the contralateral hemisphere began to attract more attention. White et al. [35] reported an acute inflammatory gene response in the contralateral hemisphere after unilateral controlled cortical impact in rats that occurred despite the absence of activated inflammatory cells, and suppression of the major inflammatory genes could prevent the progression of injury. Fury et al. [36] showed that 822 genes were significantly perturbed in the contralateral hemisphere after stroke, and some were related to metabolism, inflammation, and signal transduction. These genetic changes may mean the existence of adaptive reshaping mechanisms. In our study, combined with the severity of the injury, we found that microglia and astrocytes in the contralateral motor cortex continued to proliferate throughout the subacute phase. Some genes with increased expression are associated with well-known inflammatory signaling pathways, such as the Mapk and RAP1 pathways. Gene Set Enrichment Analysis (GSEA) showed that these genes were mainly clustered in the FoxO and Hif1 pathways, both of which play a role in the inflammatory response. Genes associated with synaptic and axon repair, such as Met, Sema3e, and Adcy9, were significantly elevated, consistent with the observed increase in synapsin-1 and PSD-95 levels.

Transcription factors are important regulators of gene activity, playing a significant role in regulating transcription and contributing to many biological functions. Therefore, identifying TF-target regulation is essential for understanding changes in transcriptional regulation [37]. Our study identified significantly upregulated TFs, including NF-KB1, STAT3, Ep300, Rela, and Jun. NF-KB1 and Rela are critical inflammatory mediators and play a vital role in cell survival. Acetylation of Rela is crucial for the proapoptotic activity of NF-KB [38,39,40]. STAT3 and Jun are associated with neuroprotection and neuron survival [41,42,43,44]. After traumatic brain injury, activation of the STAT3 signaling pathway in astrocytes actively contributes to the synaptic remodeling caused by the injury [13]. Tyzack et al. [45] reported STAT3-regulated TSP-1 expression, which promoted synaptic plasticity after motor neuron injury. Interestingly, an increase in astrocytes in the contralateral motor cortex was observed in our study. Furthermore, GSEA showed upregulation of Tgfb1, which was related to STAT3 and Jun. The Tgfb1 protein is composed of polypeptides with multiple functions and plays a vital role in the healing process of the central nervous system. Astrocytes promote an increase in cortical excitatory synapses by upregulating transforming growth factor beta1 (Tgfb1) [46,47]. These results are consistent with the increase in synapsin-1 density we observed. Therefore, we propose that damage to one side of the motor cortex triggers the proliferation of astrocytes on the other side and that these astrocytes promote synaptic development by secreting transforming growth factor β1 (Tgfb1), which is regulated by signal transducer and activator of transcription factor 3 (STAT3) and transcription factor Jun.

There are several limitations to this study. Although there is evidence that genetic modification is ongoing and widespread, examination of contralateral motor cortex changes after unilateral resection was limited to the 7-day postoperatively time point. Future research will try to monitor these contralateral changes over a longer time horizon. A significant increase in the expression of genes associated with inflammation was observed in the contralateral motor area, accompanied by glial cell proliferation. Since inflammation can have both beneficial and harmful effects, its impact on overall synaptic plasticity deserves further investigation. The current study was conducted only in male rats, which may ignore potential gender-related differences in inflammatory responses and synaptic recovery. Subsequent studies will be designed to include male and female subjects to overcome this limitation.

## 5. Conclusions

After resection of the right motor cortex, moderate contralateral changes related to activation of pathways related to inflammation and synaptic remodeling occurred. The inflammation and synaptic adaptation changes observed in the contralateral hemisphere after unilateral brain injury merit more in-depth study.

## Data Availability

The datasets presented in this article are not readily available because the data are part of an ongoing study. Requests to access the datasets should be directed to Guoyi Gao.

## Supplementary Materials

The following supporting information can be downloaded at https://www.mdpi.com/article/10.3390/brainsci15080837/s1. Figure S1: The WB results of Synapsin-1, IL-1β, IL-6, and TGF-β in the contralateral motor cortex. (A) WB bands of Synapsin-1, IL-1β, IL-6, and TGF-β. (B) In the contralateral motor cortex, the expression of Synapsin-1 increased at 7 days after traumatic brain injury (*n* = 3; *p* = 0.009). (C) In the contralateral motor cortex, the expression of IL-1β and TGF-β increased at 7 days after traumatic brain injury. However, the expression of IL-6 remained unchanged (*n* = 3, IL-1β: *p* < 0.001; TGF-β: *p* = 0.007; IL-6: *p* = 0.37). (D) In the contralateral motor cortex, the expression of PSD-95 increased at 7 days after traumatic brain injury (*n* = 3; *p* = 0.005).
