# Supplementary material for: Contralateral Structure and Molecular Response to Severe Unilateral Brain Injury
Source: Brain Sci. 2025 Aug 5;15(8):837. doi: 10.3390/brainsci15080837 (PMC12384278; doi:10.3390/brainsci15080837)
Supplement: Supplementary file 1 [file brainsci-15-00837-s001.zip › brainsci-3737250-supplementary.pdf]

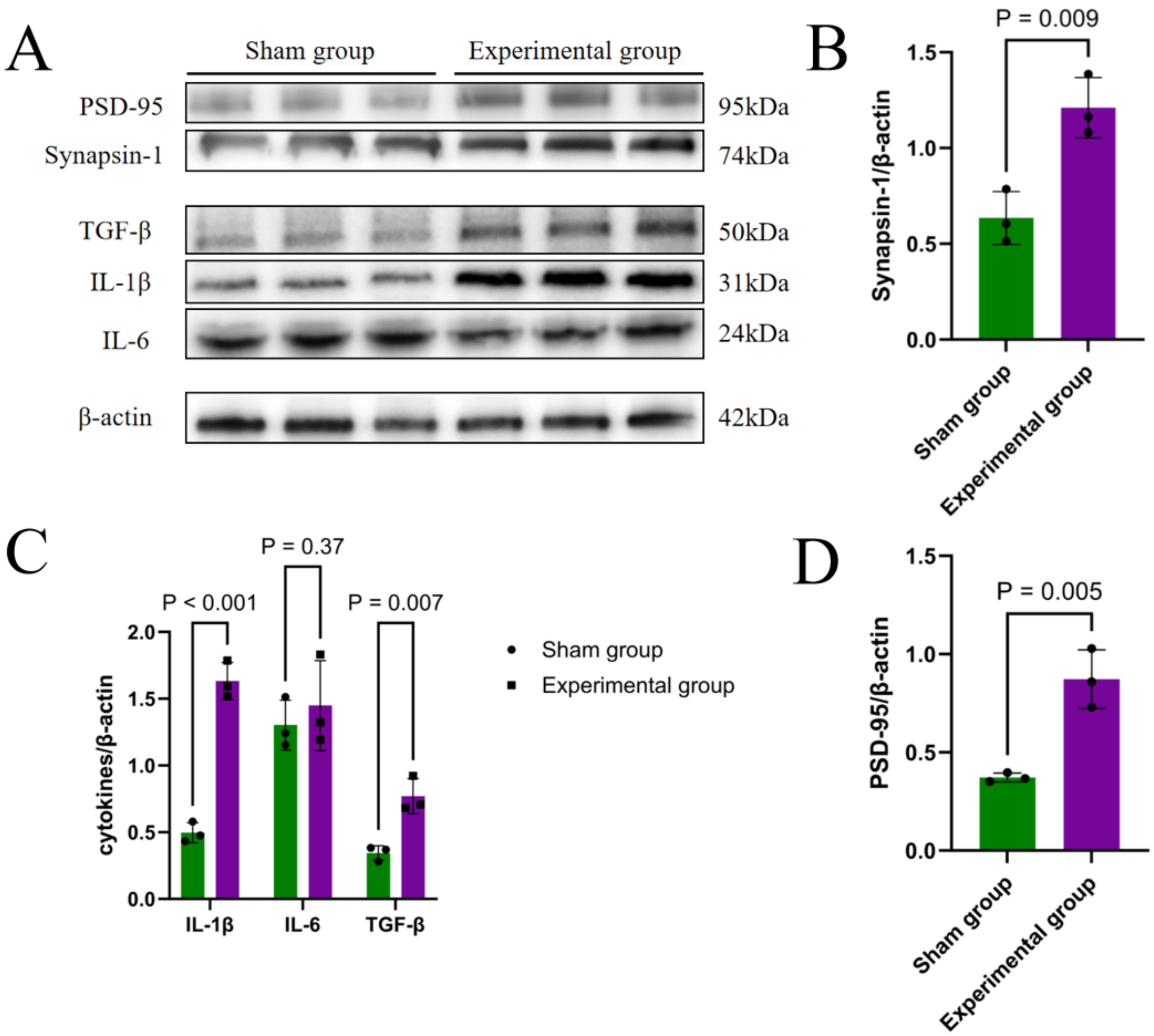

Figure S1: The WB results of Synapsin-1, IL-1β, IL-6, and TGF-β in the contralateral motor cortex. (A) WB bands of Synapsin-1, IL-1β, IL-6, and TGF-β. (B) In the contralateral motor cortex, the expression of Synapsin-1 increased at 7 days after traumatic brain injury ($n = 3$; $p = 0.009$). (C) In the contralateral motor cortex, the expression of IL-1β and TGF-β increased at 7 days after traumatic brain injury. However, the expression of IL-6 remained unchanged ($n = 3$, IL-1β: $p < 0.001$; TGF-β: $p = 0.007$; IL-6: $p = 0.37$). (D) In the contralateral motor cortex, the expression of PSD-95 increased at 7 days after traumatic brain injury ($n = 3$; $p = 0.005$).
